# Mechanical Synthesis and Calorimetric Studies of the Enthalpies of Formation of Chosen Mg-Pd Alloys

**DOI:** 10.3390/molecules29235734

**Published:** 2024-12-05

**Authors:** Adam Dębski, Magda Pęska, Julita Dworecka-Wójcik, Władysław Gąsior, Wojciech Gierlotka, Robert Chulist, Radovan Cerny, Iwona Wyrębska, Sylwia Terlicka, Marek Polański

**Affiliations:** 1 Institute of Metallurgy and Materials Science, Polish Academy of Sciences, 25 Reymonta Street, 30-059 Kraków, Poland; w.gasior@imim.pl (W.G.); rchulist@agh.edu.pl (R.C.); s.terilcka@imim.pl (S.T.); 2 Department of Functional Materials and Hydrogen Technology, Military University of Technology, 2 Kaliskiego Street, 00-908 Warsaw, Poland; magda.peska@wat.edu.pl (M.P.); julita.dworecka@wat.edu.pl (J.D.-W.); iwona.wyrebska@wat.edu.pl (I.W.); marek.polanski@wat.edu.pl (M.P.); 3 Materials Science and Engineering Department, National Dong Hwa University, Hualien 970024, Taiwan; wojtek@gms.ndhu.edu.tw; 4 Faculty of Metals Engineering and Industrial Computer Science, AGH University of Science and Technology, Al. A. Mickiewicza 30, 30-059 Krakow, Poland; 5 DQMP—Department of Quantum Matter Physics, Université de Genève, 24, Quai Ernest-Ansermet, 1211 Geneva, Switzerland; radovan.cerny@unige.ch

**Keywords:** intermetallics, enthalpy, thermodynamic properties, calorimetry, X-ray diffraction

## Abstract

Despite many years of research and continuous improvements in scientific equipment, some of the thermodynamic properties of binary systems are still unknown, or are only theoretically predicted or calculated. This situation often arises from the difficulties in preparing alloys for experimental measurements. The alloys from the Mg-Pd system, especially for the Pd-rich side, are difficult to produce, and the availability of thermodynamic data is very limited. Therefore, this paper presents calorimetric studies on the standard enthalpy of formation of alloys from the Mg-Pd system, which were prepared using mechanical alloying. Three alloys (S1, S2, and S3) were synthesized, homogenized, and subjected to X-ray diffraction (XRD) analysis to investigate their phase composition. The XRD studies showed that the alloys designated as S1 and S2 were the intermetallic phases Mg_6_Pd and Mg_0.9_Pd_1.1_, and the S3 sample was a mixture of MgPd and MgPd_3_ intermetallic phases. Their heat effects, measured by drop calorimetry, were used to calculate the values of the standard enthalpies of formation of the prepared phases. The values obtained were as follows: −27.5 ± 1.1 kJ/mol at. for the Mg_6_Pd intermetallic phase, −72.7 ± 1.0 kJ/mol at. for the Mg_0.9_Pd_1.1_ intermetallic phase, and −46.8 ± 1.5 kJ/mol at. for the alloy which was a mixture of MgPd and MgPd_3_. These data were compared with values from the existing literature on the enthalpy of formation of alloys, as well as with data calculated using Miedema’s model.

## 1. Introduction

Magnesium-based alloys, due to their low density, high relative strength, and high corrosion resistance, are highly attractive materials for a wide range of industrial and scientific applications. One of their most compelling features is their potential for hydrogen storage. Hydrides formed from magnesium alloys are considered among the most promising materials for hydrogen storage due to their high gravimetric hydrogen capacity (up to 7.6% mass for MgH_2_) and the relatively low cost of the base material. Additionally, magnesium is non-toxic and widely available. However, the practical application of magnesium for hydrogen storage is hindered by the necessity of using high temperatures and by its slow hydrogen sorption kinetics.

Research is ongoing worldwide to overcome these limitations by modifying magnesium alloys with various additives to improve their hydrogen sorption kinetics [1,2,3,4,5,6,7,8]. Among these additives, palladium and its alloys have shown significant promise due to their catalytic properties, which enhance hydrogen absorption and desorption rates. Unfortunately, the cost of palladium is so high that its use should be limited to the minimum; otherwise, such a catalytic additive will increase the price of the base material so much that the application will be unreasonable. Palladium itself is very active and reacts with hydrogen readily, but forms several intermetallic compounds (with different stabilities), some of which are known to not react with hydrogen at all in reasonable conditions [9,10,11].

Magnesium–palladium (Mg-Pd) alloys, apart from their hydrogen storage capabilities, have also shown potential in other applications, such as biomaterials, due to their excellent biocompatibility and biodegradability. Also, the use of noble metals like palladium can enhance the antibacterial effects, biocompatibility, and mechanical properties of these alloys. For that reason, it is very important to obtain systematic knowledge about the Mg-Pd system, which contains many intermetallic phases. Having this knowledge would allow for the improvement and modification of metallurgical processes, and would also enable the definition of the stabilities of some phases. In our previous works [12,13,14,15], a literature review and a justification of the research conducted in this area, with a focus on thermodynamic studies of the Mg-Pd system, were presented. Therefore, to obtain more details that will be omitted from this article, it is suggested to acquaint oneself with these previous works.

Despite these advancements, detailed information on the thermodynamic properties of these alloys, such as their enthalpy of phase formation, remains sparse. Our previous works provided insights into the thermodynamic properties of Mg-rich phases from Mg-Pd phase diagrams, but these studies were based on alloys obtained through melting and casting methods. This approach, while informative, may not fully capture the properties of materials, since chemical segregation is usually observed in cast alloys.

The preparation of intermetallic phases containing magnesium and a metal with a much higher melting point is difficult to perform using a metallurgical method, and sometimes even impossible, due to the high pressure of magnesium at elevated temperatures. Therefore, the authors set themselves the goal of investigating the possibility of producing intermetallic phases from a magnesium–palladium system using the mechanical alloying method, which is carried out close to room temperature, thus enabling the avoidance of magnesium evaporation. To confirm such a possibility, we investigated the structure and the change in the enthalpy of phase formation, and then considered this in the light of the results of previous experimental studies and theoretical calculations.

## 2. Results and Discussion

### 2.1. Phase Analysis and Microstructural Characterization

**Figure 1:**
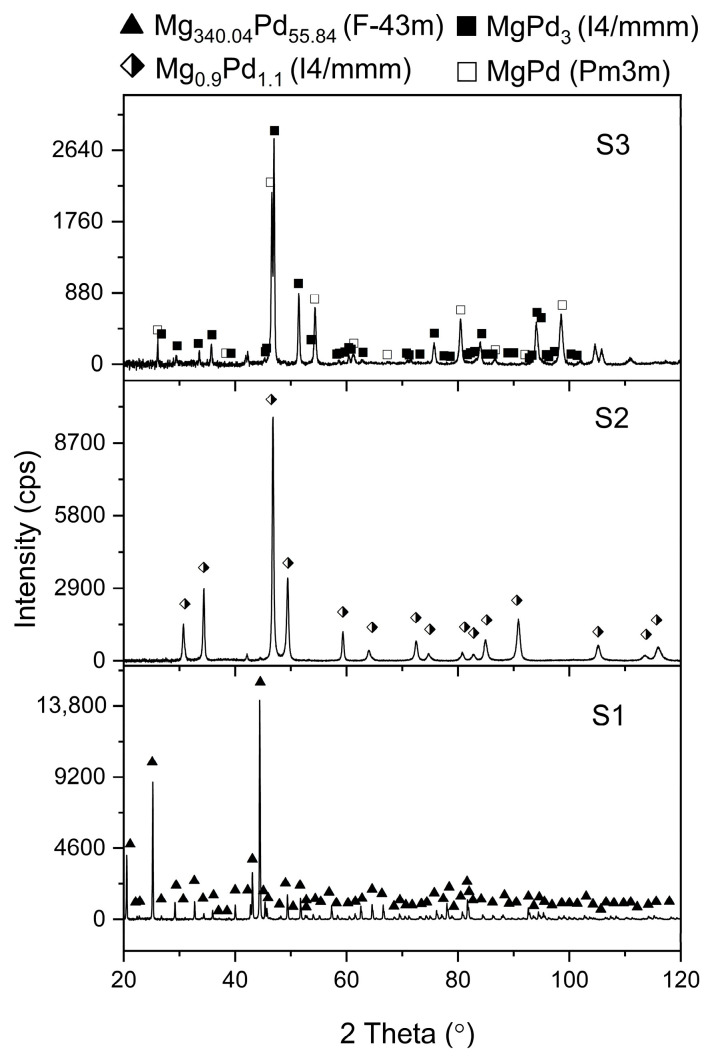
XRD patterns of Mg-Pd samples (S1–S3) directly after the mechanical alloying process with further annealing.

**Figure 2:**
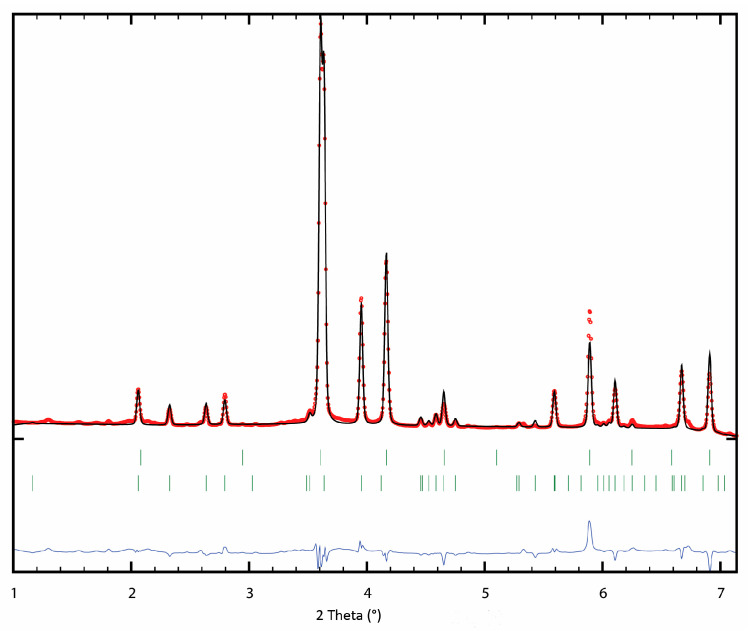
Results of Rietveld refinement using synchrotron radiation powder diffraction pattern of S3 sample. Wavelength λ = 0.014234 nm. Bragg peak ticks: MgPd (**upper**), MgPd_3_ (**lower**).

Mg-Pd alloys were obtained and then checked using the X-ray diffraction (XRD) method. The resulting diffractograms are shown in Figure 1. The XRD studies performed indicated that the alloys designated as S1 and S2 could be assumed to be the intermetallic phases Mg_6_Pd and Mg_0.9_Pd_1.1_. In Eisheh’s PhD Dissertation [16], the Pd-rich part of the phase diagram was investigated with electromotive force measurement and diffusion couple analysis. In his work, the Mg_3_Pd_5_ phase was presented instead of the Mg_0.9_Pd_1.1_ phase. In contrast, in our work, the alloy designated as S3 showed the existence of two MgPd_3_ phases known from the literature: *I*4/*mmm* and *Pm*3*m*. A Rietveld refinement, which used a synchrotron radiation powder pattern, converged to the composition MgPd_3_ for the former phase; however, for the latter phase, it converged to the composition ~MgPd. Contrary to the data from the literature, the Wyckoff site 0, 1/2,1/2 of the space group *Pm*3*m* was refined as a mixed occupancy site of Mg_0.36_Pd_0.61_. The Rietveld plot is presented in Figure 2. Therefore, in the continuation of this work, the alloy denoted as S3 will not be treated as an intermetallic phase, but as an alloy whose concentration corresponds to a mixture of 80(1) wt. % of MgPd and 20(1) wt. % of MgPd_3_ intermetallic phases.

### 2.2. Calorimetric Studies

The heat of solution values (limiting partial enthalpies of the solutions) of Mg and Pd in liquid tin or liquid aluminum, presented in Table 1, are taken from our previous study [13,14]. Moreover, the thermochemical data of the metals were calculated using Pandat 2013 [17] (Pan SGTE database based on the original SGTE v4.4 database [18]). The heat effects, as well as the values of the standard enthalpies of formation of the measured phases determined from them, are shown in Table 2.

**Figure 3:**
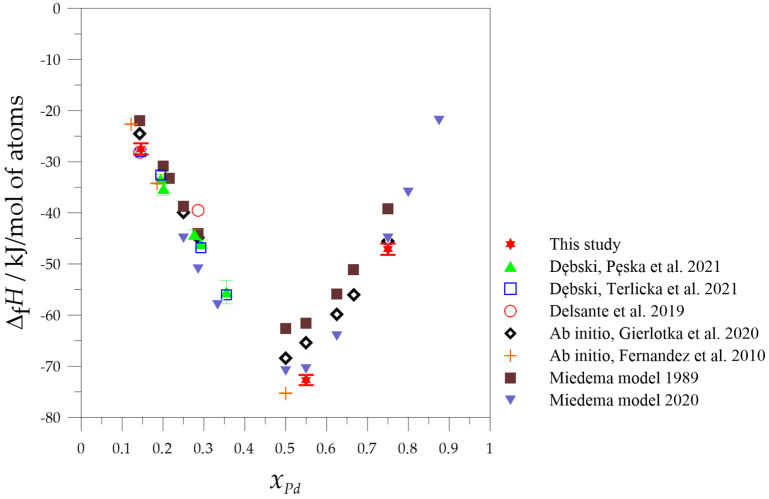
Comparison of the experimental and calculated values of the standard formation enthalpies for intermetallic phases and alloys from the Mg-Pd system (solution method: this study [13,14], direct reaction method [19]), with ab initio calculations [12,20] and the Miedema model [21,22].

A comparison of the formation enthalpies of the investigated alloys obtained in this study with the data from the literature is presented in Figure 3.

As seen in Figure 3, the obtained formation enthalpy of the Mg_6_Pd intermetallic phase is in good agreement with the data from the literature [13,14,19]. The values measured for the samples produced by the metallurgical method [13,14] and by the mechanical alloying method (this work) are almost identical. Since the calorimetric studies were conducted using the same method and under identical conditions (high-purity argon), this indicates that the material must have been characterized by the same structure and chemical composition in the other studies. Therefore, the preparation of the Mg_6_Pd intermetallic phase can also be conducted using the mechanical synthesis method, which, due to the low temperature of the process, does not cause magnesium loss in comparison with the metallurgical method.

Since the structural studies conducted for the Mg_0.9_Pd_1.1_ phase produced by mechanical alloying fully confirmed its structure, it should also be expected that other phases with a Mg concentration higher than 0.45 molar fraction can be obtained by this method, and that the results of the calorimetric studies will be very similar to those for phases with an identical structure but produced by other methods. Therefore, in our opinion, the use of mechanical alloying seems to be the best solution for obtaining Mg-Pd alloys. This is related to the fact that obtaining these alloys by melting and casting is difficult, mainly due to differences in melting temperatures and the fact that magnesium is characterized by a high vapor pressure. The melting and casting mentioned above may lead to differences in the assumed structural and chemical composition, due to the evaporation of one of the components. Another problem is the gravitational segregation that occurs during solidification due to differences in density. This is why mechanical synthesis was used to prepare these alloys, mainly because all the reactions occur in a solid state, and thus all the above-mentioned issues related to the liquid state can be avoided.

According to the models presented in the literature [23], the mechanical synthesis process consists of two stages. In the first stage, the particles of one component are mixed with the particles of the second component and strain hardened. At this stage, the mixture is homogenized, which ensures contact between the particles of different elements. This contact provides a shortened path for the second stage, which is diffusion. Depending on the type of components used, the third stage may occur—the nucleation and formation of the new intermetallic phase (as in this case). The shear and impact forces that occur during milling lead to the introduction of many crystal lattice defects, which can affect the free energy of the resulting phases, and lead to the formation of metastable phases [24]. Analyzing Figure 3, it can be noted that the obtained results of the standard enthalpy of formation of the phases from the Mg-Pd system, measured by solution calorimetry, showed better compatibility with those obtained by ab initio calculations compared to those calculated by the Miedema model [21], in which the enthalpy of formation of the intermetallic phase is determined from the following equation:

(1) $$\Delta_{f}H=\frac{x_{A}V_{A\left(\mathrm{alloy}\right)}^{\frac{2}{3}}\cdot f_{B}^{A}[-{\left(\Delta \Phi^{*}\right)}^{2}+\frac{Q}{P}(\Delta n_{\mathrm{ws}}^{\frac{1}{3}}{)}^{2}-\frac{R}{P}]}{\left[{\left(n_{\mathrm{ws}}^{A}\right)}^{\frac{-1}{3}}+(n_{\mathrm{ws}}^{B}{)}^{\frac{-1}{3}}\right]}$$

where x_A_ represents the mole fraction of component A in the binary alloy; $V_{A\left(\mathrm{alloy}\right)}^{\frac{2}{3}}$ is the molar volume of element A in the alloy; $f_{B}^{A}$ is the fraction of atomic cells of A atoms surrounded by B atoms; $\Delta \Phi^{*}$ = $\phi_{A}-\phi_{B}$ is the chemical-potential difference between the pure elements A and B; $n_{\mathrm{ws}}^{A}$ and $n_{\mathrm{ws}}^{B}$ are the densities of electrons at the Wigner-Seitz cell boundary; and R, P, and Q are empirical constants, determined by Miedema et al. [21]. The difference between the values calculated from Miedema’s model in [21,22] results from the difference in the assumed value of the R/P parameter for magnesium. According to the analysis in [22], an R/P value of 0.7 is the optimal value of the parameter for Mg. It causes the calculated values of the enthalpy of formation to be more negative than those calculated for a Mg parameter value of 0.4 [21].

As can be seen from the comparison of experimental data [13,14,19] and ab initio calculations [12,20], the agreement between these is satisfactory, and sometimes the values differ in terms of their estimated error. The conclusion resulting from this observation may be that in many cases, the results of ab initio calculations, especially the changes in the enthalpy of intermetallic phase formation, can replace very difficult to perform experimental studies and use these data for other purposes, e.g., calculating phase diagrams. However, it should always be borne in mind that theoretical calculations use different models and methods for this purpose, and the results obtained from them also differ significantly, which can also be observed in the case of the Mg-Pd system (Figure 3). Therefore, in the authors’ opinion, experimental data should always be preferred if possible, if obtained using a reliable and appropriate method for studying energy effects, phase preparation, and structure verification.

## 3. Materials and Methods

**Figure 4:**
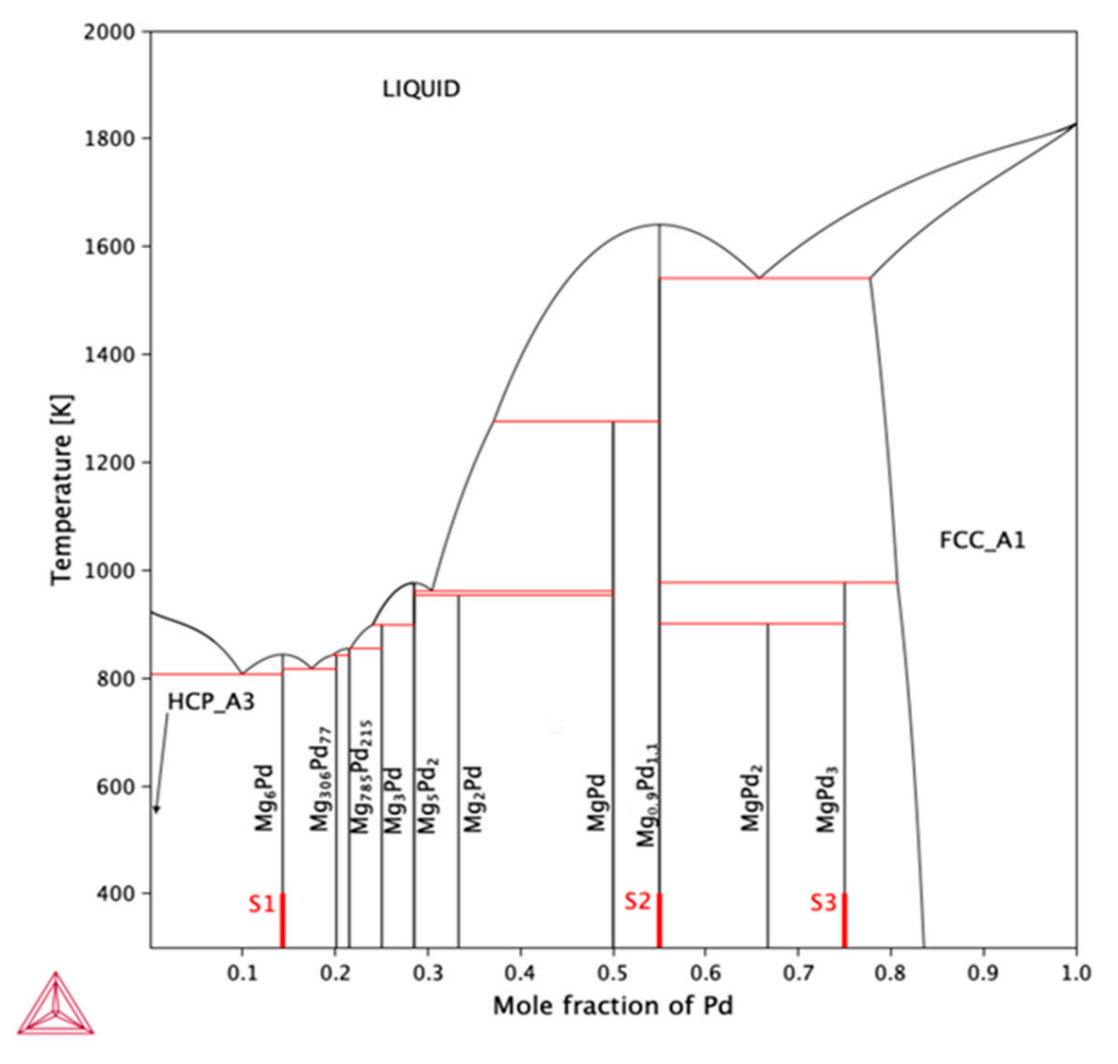
Phase diagram of the Mg-Pd system together with the chemical compositions of the obtained samples (S1–S3). Thermodynamic parameters taken from [15].

**Figure 5:**
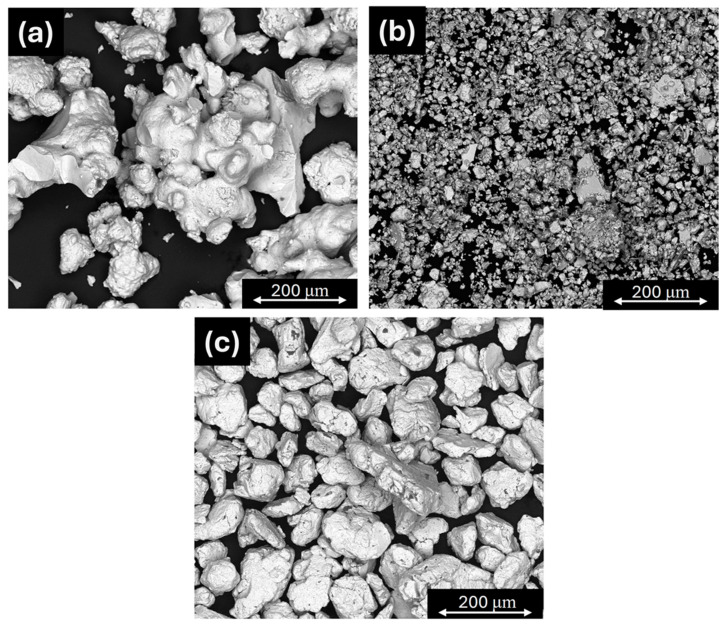
Morphology of powder particles after mechanical synthesis and annealing for alloys (**a**) S1 (Mg_6_Pd), (**b**) S2 (Mg_0.9_Pd_1.1_), and (**c**) S3 (MgPd_3_, MgPd).

Information on the purity and manufacturer of the materials used to produce the intermetallic phases of the Mg-Pd system is presented in Table 3. As mentioned earlier, the intermetallic phases were produced by mechanical alloying. Milling was performed in a planetary ball mill (Fritsch Pulverise 7, Idar-Oberstein, Germany), and the prepared Mg-Pd alloys are presented in Table 4 and Figure 4 (S1–S3). The total weight of the S1 and S2 samples was 2 g, and in the case of S3, it was 3 g, due to the small volume of the sample. The powders were weighed to a precision of 0.0001 g. Then, the prepared samples were ball-milled in a 20 mL stainless steel vial (S1, S2) and a Si_3_N_4_ vial (S3) using 10 bearing steel balls (S1, S2) and a Si_3_N_4_ ball (S3), each 10 mm in diameter. The preparation of powders was performed in a glove box (Mbraun LabMaster, Munich, Germany) with a high-purity environment of constantly purified argon (<1 ppm O_2_ and H_2_O). Next, each sample was milled at 600 rpm for 1 h (S1, S2) and 5 h (S3). After the ball-milling process, the as-milled powder was enclosed in a specifically designed stainless steel reactor with a tight seal (copper sealing gasket). To minimize the risk of oxidation of the as-milled powders, the furnace used for the annealing process was located in a LabMaster (MBraun) glovebox. The homogenization conditions of the prepared alloys are shown in Table 5. After the annealing process, the prepared samples were subjected to XRD phase analysis. For this purpose, the X-ray powder diffractometer (Ultima IV Rigaku, Tokyo, Japan), equipped with a cobalt anode lamp (λ = 1.78 Å), was used. The measurements were performed with the operating parameters of 40 mA, 40 kV, and a scanning speed no higher than 1°/min, and with the use of cross beam optics (CBO), parallel-beam geometry, and a fast linear counter (Detex Ultra, Rigaku, Tokyo, Japan), which resulted in patterns with very low Kβ levels. The analysis of the obtained X-ray diffractions was performed using PDXL2 2.8.4.0 software. Additionally, in the case of the S3 sample, X-ray diffraction synchrotron studies were performed. These measurements were conducted employing a beam line P07 (87.1 keV, λ = 0.014234 nm) at DESY Hamburg. A beam with a cross-section of 0.8 × 0.8 mm^2^ was applied using transmission geometry, giving the diffraction information for large sample volumes (0.8 × 0.8 × 2 mm^3^). To avoid the effect of texture, the samples were rotated 180 degrees about the ω axis. Such a procedure allows for the collection of all the diffraction patterns on one single image, which significantly facilitates Rietveld refinement for bulk samples. This pattern was used for the Rietveld refinement of the intermetallic phases in sample S3; the program Fullprof.2k, Version 5.80 [25] was used. Next, high-resolution field emission scanning electron microscopy (FEI Quanta 3D, Hillsboro, OR, USA) was used to examine the morphology of the obtained powders after the mechanical alloying process. Figure 5 shows the morphology of powder particles after mechanical synthesis and annealing. The BSE observations show a particle appearance that is typical of ball-milled samples.

The calorimetric studies were conducted in a protective argon atmosphere with the use of a Setaram MHTC 96 line evo drop calorimeter (Setaram Instrumentation—KEP technologies, Caluire, France). The calorimetric study procedure was analogous to that in our previous articles [13,14], and the intermetallic phase samples were dissolved in a metal bath in alumina crucibles.

The enthalpy of formation (Δ_f_*H*) values of the measured phases were calculated from the differences in their heat effects, which were investigated by heating the samples from room temperature (298 K) to the measurement temperature, and observing the dissolution of the studied phases and their components in a metallic bath (in liquid tin or aluminum). The Δ_f_*H* values were computed using the following equation:

(2) $$\Delta_{f}H=x_{\mathrm{Mg}}\Delta H_{\mathrm{Mg}}^{0}+x_{\mathrm{Pd}}\Delta H_{\mathrm{Pd}}^{0}-\Delta H_{x_{\mathrm{Mg}}x_{\mathrm{Pd}}}^{0}$$

where Δ_f_*H* is the enthalpy of formation of the measured phase; $x_{\mathrm{Mg}}$ and $x_{\mathrm{Pd}}$ are the mole fractions of the components, respectively; and $\Delta H_{\mathrm{Mg}}^{0}$, $\Delta H_{\mathrm{Pd}}^{0}$, and $\Delta H_{x_{\mathrm{Mg}}x_{\mathrm{Pd}}}^{0}$ are the heat effects accompanying the dissolution of one mole of the components Mg, Pd, and the phases in the aluminum bath, respectively.

## 4. Conclusions

This paper presented experimental data of the standard enthalpy of formation of selected intermetallic phases of the Mg-Pd system, which were measured using the solution calorimetry method. A reactive milling method was used to produce the above alloys, which enables the preparation of intermetallic phases, the formation of which is very difficult or even impossible using foundry methods, because of the high vapor pressure of Mg. Before taking calorimetric measurements, the samples were homogenized and then subjected to XRD measurements for verification. The lowest value of the standard enthalpy of formation, equal to −72.7 ± 1.0 kJ/mol at., was obtained for the Mg_0.9_Pd_1.1_ phase. Furthermore, the obtained value of the formation enthalpy of the Mg_6_Pd intermetallic phase agreed with the earlier data. The obtained results of the standard enthalpy of formation of the phases from the Mg-Pd system, measured by solution calorimetry, showed a stronger correlation with those obtained by ab initio calculations than with those calculated by the Miedema model.

It should be noted that the study of the enthalpy of intermetallic phase formation presented in this article, based on two different methods of obtaining the phases, is the first work that indicates the possibility of preparing high-melting phases created from metal alloys in which one component has high vapor pressure, which prevents the ability to obtain intermetallic phases and solid alloys using the melting (metallurgical) method. In addition, it has been shown that mechanical synthesis with a subsequent homogenization process is an excellent method of obtaining intermetallic phases, in the case of alloys with a metal of high vapor pressure. It seems that it can also be used to obtain high-melting intermetallic phases from metals with a high vapor pressure.

## Figures and Tables

**Table 1:** The heat of solution values (limiting partial enthalpies of the solutions) of Mg and Pd in liquid tin or liquid aluminum.

Bath	Metal	Temperature [K]	Limiting Partial Enthalpy of Solution $\Delta_{sol}{\bar{H}}_{Mg\left(l\right)\;}^{\infty }$ [kJ/mol at.]	References
Sn	Mg	736	−33.03	[13]
	Pd		−126.3	
Al	Mg	1033	−8.6	[14]
	Pd		−186.8	

**Table 2:** The heat effects Δ*H*^ef^ and formation enthalpies Δ_f_*H* of alloys and intermetallic phases from the Mg-Pd system.

Sample	T [K]	Sample No.	Δ*H*^ef^ [kJ/mol at.]	Δ_f_*H* [kJ/mol at.]
Dissolution in liquid tin. Temperature of the Sn bath: 733 K.				
S1 (Mg_6_Pd)	298	1	2.4	−28.5
		2	1.0	−27.1
		3	1.7	−27.7
		4	0.8	−26.9
		Average	1.5	−27.5
		Standard error	1.1	1.1
S2 (Mg_0.9_Pd_1.1_)	298	1	10.6	−73.2
		2	10.1	−72.8
		3	9.2	−71.8
		4	10.4	−73.1
		Average	10.1	−72.7
		Standard error	1.0	1.0
Dissolution in liquid aluminum. Temperature of the Al bath: 1033 K.				
S3 (MgPd_3_, MgPd)	298	1	−64.6	−45.8
		2	−62.5	−47.9
		3	−62.9	−47.5
		Average	−63.4	−47.1
		Standard error	1.1	1.1

**Table 3:** Specifications of the applied materials.

Chemical Name	Source	Purity [Mass%]
Magnesium powder	Sigma Aldrich, Poznań, Poland	>99%
Palladium wire	Safina a.s., Vestec, Czech Republic	99.95
Argon	Pioniergas, Krakow, Poland	99.9999

**Table 4:** Composition of obtained Mg-Pd alloys.

No.	Alloys (Phases)	Magnesium		Palladium	
		At.%	Mass%	At.%	Mass%
1	S1 (Mg_6_Pd)	85.71	57.81	14.29	42.19
2	S2 (Mg_0.9_Pd_1.1_)	45.01	15.75	54.99	84.25
3	S3 (MgPd_3_, MgPd)	24.99	7.07	75.01	92.92

**Table 5:** Homogenization conditions of the prepared alloys.

No.	Alloys (Phases)	Annealing Temperature [K]	Annealing Time [h]
1	S1 (Mg_6_Pd)	773	5
2	S2 (Mg_0.9_Pd_1.1_)	773	5
3	S3 (MgPd_3_, MgPd)	973	5

## Data Availability

The data that support the findings of this study are available from the corresponding author, [A.D.], upon reasonable request.
